# Molecularly Imprinted Nanoparticle Ensembles for Rapidly Identifying *S. epidermidis*

**DOI:** 10.3390/s23073526

**Published:** 2023-03-28

**Authors:** Chularat Hlaoperm, Wisnu Arfian A. Sudjarwo, Jakob Ehrenbrandtner, Endre Kiss, Giorgia Del Favero, Kiattawee Choowongkomon, Jatuporn Rattanasrisomporn, Peter A. Lieberzeit

**Affiliations:** 1 University of Vienna, Faculty for Chemistry, Department of Physical Chemistry, Waehringer Strasse 42, A-1090 Wien, Austria; 2 Center for Advanced Studies for Agriculture and Food, Kasetsart University Institute for Advanced Studies, Kasetsart University, Bangkok 10900, Thailand; 3 University of Vienna, Faculty for Chemistry, Doctoral School of Chemistry, Waehringer Strasse 42, A-1090 Vienna, Austria; 4 University of Vienna, Faculty for Chemistry, Core Facility Multimodal Imaging, Waehringer Strasse 38, A-1090 Vienna, Austria; 5 University of Vienna, Faculty for Chemistry, Department of Food Chemistry and Toxicology, Waehringer Strasse 38, A-1090 Vienna, Austria; 6 Department of Biochemistry, Faculty of Science, Kasetsart University, Bangkok 10900, Thailand; 7 Department of Companion Animal Clinical Sciences, Faculty of Veterinary Medicine, Kasetsart University, Kamphaeng Saen Campus, Nakhon Pathom 73140, Thailand

**Keywords:** AFM, molecularly imprinted polymers, nanoparticles, *Staphylococcus epidermidis*

## Abstract

*Staphylococcus epidermidis* (*S. epidermidis*) belongs to methicillin-resistant bacteria strains that cause severe disease in humans. Herein, molecularly imprinted polymer (MIP) nanoparticles resulting from solid-phase synthesis on entire cells were employed as a sensing material to identify the species. MIP nanoparticles revealed spherical shapes with diameters of approximately 70 nm to 200 nm in scanning electron microscopy (SEM), which atomic force microscopy (AFM) confirmed. The interaction between nanoparticles and bacteria was assessed using height image analysis in AFM. Selective binding between MIP nanoparticles and *S. epidermidis* leads to uneven surfaces on bacteria. The surface roughness of *S. epidermidis* cells was increased to approximately 6.3 ± 1.2 nm after binding to MIP nanoparticles from around 1 nm in the case of native cells. This binding behavior is selective: when exposing *Escherichia coli* and *Bacillus subtilis* to the same MIP nanoparticle solutions, one cannot observe binding. Fluorescence microscopy confirms both sensitivity and selectivity. Hence, the developed MIP nanoparticles are a promising approach to identify (pathogenic) bacteria species.

## 1. Introduction

*Staphylococcus epidermidis* (*S. epidermidis*) is one of the most common Gram-positive bacteria species found on mucous membranes of human skins and animals living closely together with humans, such as cats and dogs. It can specifically adhere to adhesin receptors of host tissue surfaces [1,2,3]. Based on previous medical reports, *S. epidermidis* infections occur at a wide range of medical devices, intravascular catheters, prosthetic joints, artificial heart valves, and cerebrospinal fluid shunts. This even may lead to the death of hospital patients [4,5,6]. Those result from infection with a methicillin-resistant strain containing a slime capsule as a natural barrier. This also prevents other antibiotics from entering the cell, including rifamycin, fluoroquinolones, gentamicin, tetracycline, chloramphenicol, erythromycin, clindamycin, and sulfonamides [7,8]. 

Antimicrobial susceptibility in companion animals is a potential public health issue since Staphylococci isolated from cats and dogs had methicillin-resistant patterns similar to those of humans [9]. Current methods detecting *S. epidermidis* infection are antimicrobial susceptibility test [10], cultivation dependent methods [11], Gram-stain [12], Christensen’s test-tube method [13], and polymerase chain reaction (PCR) [14]. However, those methods are frequently time-consuming, labor intensive, and incur high instrument and maintenance costs. 

In a first step to overcome these limitations, herein, we synthesized molecularly imprinted polymer (MIP) nanoparticles grown directly on the cell walls of *S. epidermidis* [15,16], mimicking the solid phase synthesis approach. During polymerization, the oligomer interacts with functionalities on the surface [17]. Previous studies revealed that MIPs against whole cells, such as bacteria and viruses, are suitable to distinguish between different microorganism types and strains [18]. However, whole-cell templates may have limited usability due to their large size, chemically complex surface, and comparably weak interactions with the MIP [19]. Generally speaking, smaller species, such as amino acids, peptides, and nucleic acids, have led to more straightforward synthesis of stable and selective MIPs [20,21]. Pan, Xue, and co-workers have prepared MIPs to recognize protein A on surfaces of *S. aureus* [22]. These reports demonstrate that preparing MIPs in situ for cell membrane components improves selectivity and specific recognition of whole cells.

In this work, we prepared MIP nanoparticles using “solid phase” synthesis by applying *S. epidermidis* as the template species and growing the MIP NPs on their surfaces in an undirected manner (in a kind of “shotgun approach”). The original solid phase approach to synthesize mimics of monoclonal antibodies goes back to the groups of S. Piletsky [23] and K. Haupt [24] Basically, it comprises of immobilizing the target template on a solid support, usually glass beads or silica gel. After that, one adds a suitable monomer solution to generate MIP nanoparticles in situ on the solid support. Washing sequentially with cold water followed by hot water allows for obtaining fractions containing high-affinity particles. Bacteria cell walls contain a wide variety of compounds on their cell surfaces that differ between species [25]. Hence, it should be possible to distinguish bacteria species from each other by exposing them to an ensemble of MIP nanoparticles synthesized utilizing a specific species as the template, and thus targeting its specific lipids, saccharides, peptides, and proteins [25]. Therefore, such MIP nanoparticle ensembles would make it possible to establish a rapid test that identifies a certain species without the need of extracting and amplifying genetic material. A previous study has reported a somewhat similar MIP nanoparticle synthesis for cancer cell proteomics [26]. However, that article does not use MIP ensembles for identifying cell species.

To assess whether this is a feasible way for achieving rapid identification, we have prepared MIPs nanoparticles to recognize the surface of *S. epidermidis.* Scanning Electron Microscopy (SEM), AFM, and Fluorescent microscopy served to investigate and visualize efficiency, analyte rebinding, and selectivity. For this purpose, we compared the interaction between MIP nanoparticles and *S. epidermidis* to other bacteria, namely *Escherichia coli* (*E. coli*) and *Bacillus subtilis* (*B. subtilis*). 

## 2. Materials and Methods

### 2.1. Bacteria Culture 

*S. epidermidis*, *E. coli,* and *B. subtilis* were freshly cultured for 24 h at 37 °C in lysogeny broth containing 10 g/L proteose peptone, 5 g/L NaCl, 1 g/L D-glucose monohydrate, and 5 g/L yeast extract. The cell suspensions were centrifuged at 1900× *g* for 10 min and washed twice under sterile conditions with autoclaved distilled water before use. 

### 2.2. Immobilizing S. epidermidis 

**Figure 1:**
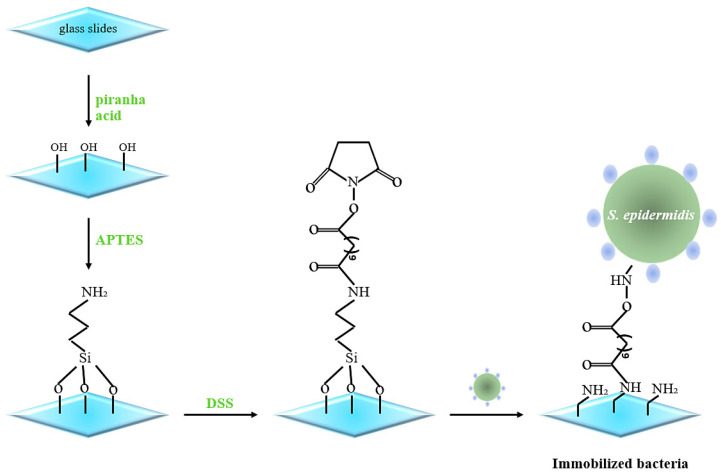
Schematic representation of immobilizing *S. epidermidis* on glass surfaces.

Glass slides (≈1.5 × 1.5 cm) were cleaned with ethanol in an ultrasonic bath for 10–12 min and, after that, immersed into Piranha acid solution (1:5 = H_2_O_2_:H_2_SO_4_ *v*/*v*) for 30 min to generate –OH groups on the surface, rinsed with water three times, and dried. After activation, the glass slides were incubated in 3-aminopropyl-triethoxysilane (APTES) 0.05% in toluene in the dark for 2 h, then washed with toluene. Afterwards, they were immersed in 9 mM disuccinimidyl suberate (DSS) and incubated for 1 h, then washed in DMSO two times to remove excess DSS. Bacteria (*S. epidermidis*, *B. subtilis* and *E. coli*) were dropped onto the center of modified glass plates and incubated at 37 °C overnight. One day later, the glass slides were washed with water five times to remove non-immobilized bacteria and left to dry. Finally, we blocked the free linker with 10 mM ethanolamine for 30 min followed by washing five times in water (Figure 1). 

### 2.3. MIP Nanoparticle Synthesis

**Figure 2:**
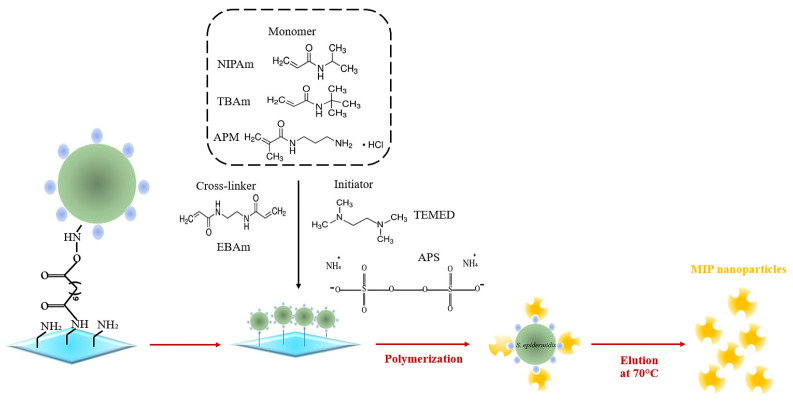
Schematic of MIP nanoparticle synthesis for *S. epidermidis*.

We used three different monomer compositions: Recipe 1 contained N-Isopropyl acrylamide (NIPAm 74.9 mg; 0.66 mmol), and Ethylene bisacrylamide (EBAm 5.6 mg; 0.06 mmol) as the crosslinker. Recipe 2 contained NIPAm (61 mg; 0.54 mmol), N-tert-butyl acrylamide (TBAm 7.7 mg; 0.06 mmol), and EBAm 5.6 mg; 0.06 mmol. Recipe 3 comprised NIPAm (61 mg; 0.54 mmol), TBAm (7.7 mg; 0.06 mmol), N-(3-aminopropyl) methacrylamide hydrochloride (APM 10.7 mg; 0.06 mmol), and EBAm (5.6 mg; 0.06 mmol). Monomers and crosslinker were mixed in 500 µL ethanol then sonicated for 10 min. Afterwards, the mixture was degassed in a water bath (37 °C; 10 min). The glass slides containing immobilized *S. epidermidis* were placed in a reaction vial using double-sided adhesive tape. In total, 15 mL of the monomer solution was then poured into the vial, followed by removing oxygen by purging with argon for 5 min. *N,N,N′,N*′-tetramethylethylenediamine (TEMED; 200 µL in 800 µL ethanol) and ammonium persulfate (APS; 200 mg in 800 µL water) were added until the solution turned opaque followed by polymerizing at 37 °C for 3 h. To extract MIP nanoparticles, the slides were washed three times with water at 37 °C (around 50 mL), followed by washing two times with water at 70 °C (around 50 mL). MIP nanoparticles were then characterized by AFM and SEM. These MIP nanoparticles were stored at 4 °C (Figure 2). For synthesizing non-imprinted particles (NIP), we carried out the same steps without immobilizing bacteria.

### 2.4. MIP Nanoparticle Rebinding

Immobilized bacteria on glass slides were then assessed using AFM, SEM, and fluorescence microscopy. The former two techniques served to record different morphologies of bacteria before and after rebinding with MIP nanoparticles; the latter to obtain slightly more (semi-) quantitative insight into binding.

### 2.5. Assessing Surface Roughness by AFM Imaging

Briefly, the morphologies of bacteria were characterized by tapping mode in air. The surface roughness of the bacterial cell surface was calculated using the data generated by AFM height images following previously published routines on a Bruker Nanoscope 8 system [1,27,28]. Bacterial cell surfaces and shapes were estimated using Gwyddion software after applying a mean filter to the raw images. Using the following equations, the surface roughness of a selected area of this flattened image was calculated from the standard deviation in the height image. The average roughness (Ra), and root-mean-square roughness (RMS or Rq), as shown in Equation (1), were utilized to characterize the surfaces of different samples.

(1) $$R_{rms}=\sqrt{\sum_{i=1}^{n}\frac{{\left(Z_{i}-Z_{m}\right)}^{2}}{N-1}}$$

$$R_{a}=\frac{1}{N}\sum_{i-1}^{N}\left|Z_{i}\right|$$

where N is the total number of data points, z_i_ is the height of the i-th point, and z_m_ is the mean height.

### 2.6. Statistical Analysis

Data were taken in duplicate from three independent cultures of each studied group. Analysis of variance (ANOVA) followed by independent *t*-test is a statistical procedure that compares the averages/means of two independent or unrelated groups to determine significant differences between the surface roughness of bacteria incubated in the presence and absence of MIP nanoparticles, the filter of roughness based on Gwyddion software. The difference is significant at a *p*-value < 0.005, non-significant at *p* > 0.005.

### 2.7. Fluorescence Imaging

Cells were stained with a fluorescence label by using the MemBrite Fix 680/700 cell surface staining kit purchased from Biotium. In brief, the first step comprised of washing immobilized bacteria several times with autoclaved milliQ water. Then, we prepared the 1× pre-staining solution by adding 1 µL of the pre-staining solution 1000× to 1 mL PBS (pH = 7.0). After that, we covered the bacteria with 10 µL of that solution and incubated for five minutes at 37 °C in a drying cabinet, followed by rinsing the surfaces several times with milliQ water. For actual staining, we added 20 µL anhydrous DMSO to the staining vial, added 1 µL of this 1000× solution to 1ml PBS (pH = 7.0), and transferred 10 µL of the resulting mix onto the bacteria surfaces. After incubating for five minutes at 37 °C in the drying cabinet, we rinsed the surface several times with milliQ water.

To stain nanoparticles, we used tetramethyl rhodamine isothiocyanate (TRITC) purchased from Sigma Aldrich. First, we dissolved 10 mg TRITC in 1 mL PBS (pH = 7.0). Then, we overlaid 4 g glass beads containing both bacteria and MIP beads (after synthesis) with a solution containing 15 µL TRITC concentrate (as prepared) in 2 mL PBS. After incubating in the dark at room temperature overnight, we rinsed several times with cold autoclaved milliQ water to remove excess dye and low-affinity MIP nanoparticles. In the last step, we eluted labelled high-affinity MIP NPs by rinsing with 15 mL autoclaved milliQ water at 70 °C. The dye binds to the primary amino groups of APM, which are present on the particle surface.

Sample slides were fixed with ROTI-Fix Spray Fixative (Carl Roth, Karlsruhe, Germany) and mounted using Vectashield HardSet mounting medium (Vector Laboratories, Inc., Newark, CA, United States) according to the manufacturer’s guidelines, covered with a glass coverslip and sealed. Fluorescence imaging took place on a Zeiss LSM710 Elyra PS.1 instrument equipped with an alpha Plan-Apochromat 100X/1.46 oil DIC objective (Carl Zeiss AG, Oberkochen, Germany). Laser-scanning confocal images were acquired with the software ZEN 2012 Black edition (Carl Zeiss AG) at 561 nm excitation/566–669 nm emission wavelengths for TRITC and at 633 nm excitation/671–759 nm emission wavelengths for MemBrite Fix 680/700. For quantitative image analysis, all bacteria images were recorded at the same microscope settings. Then, we opened them in a software (Fiji release 2.9.0; https://imagej.net/software/fiji/; accessed on 14 December 2022) and calculated the mean grey value at five positions each for bacteria and background. For that purpose, we marked bacteria with an ellipse and background areas with squares of roughly similar size to define the areas for calculation.

## 3. Results

### 3.1. MIP Characterization by SEM and AFM

**Figure 3:**
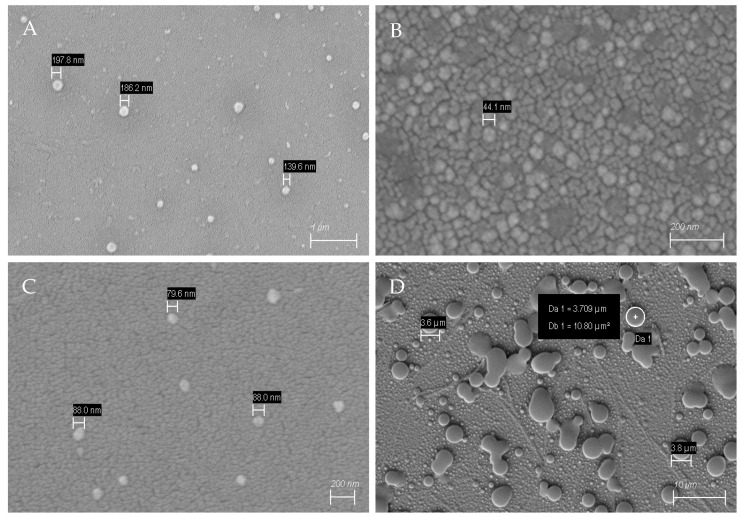
SEM of MIP and NIP nanoparticles revealing spherical shape. Their diameters are different; (**A**–**C**) are MIP nanoparticles resulting from protocol 1, 2, and 3, respectively. They are around 70–200 nm in diameter. Non-imprinted nanoparticles (**D**) are around 3.6–3.8 µm in diameter.

The scanning electron micrographs in Figure 3 show the morphologies of MIPs to confirm successful MIP nanoparticle synthesis. The amounts of monomers were varied to observe their effect of both zeta potential and nanoparticle sizes, respectively. Table 1 summarizes the protocols, zeta potentials, sizes, and yields of high-affinity particles relative to the amount of monomers used. 

We kept molar ratios of NIPAm to other monomers at 11 + 1, 5 + 1, and 3 + 1 in protocol 1, 2, and 3, respectively. From Table 1, it is evident that adding APM to the monomer mixture shifts nanoparticles zeta potentials to positive values. Indeed, one can expect this to affect the interaction between the MIP and bacteria: the latter reveal negative charge in aqueous solution. The shapes and sizes of nanoparticles were assessed using SEM as seen in Figure 3.

**Figure 4:**
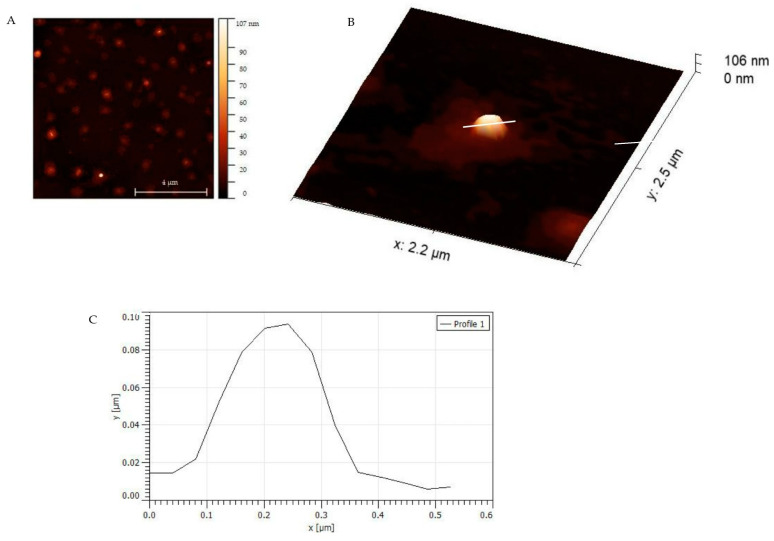
AFM characterization of MIP nanoparticles; (**A**,**B**) are AFM height images of MIP nanoparticles at a different scale have sizes around 70–107 nm. (**C**). Topographic profile obtained from height image of AFM.

SEM images show spherical MIP particles for all three monomer compositions (see Figure 3). Their diameters depend on the synthesis protocol, though: MIP of composition 1 are around 186–199 nm in diameter, which makes them the largest of all MIP nanoparticles (Figure 3A). Adding TBAM (Figure 3B; composition 2), leads to individual particle sizes around 44 nm, i.e., the smallest particles observed. However, they tend to cluster. Recipe 3 leads to globular MIP around 79–88 nm in diameter. Finally, non-imprinted nanoparticles (NIP) are around 3–4 µm in size (Figure 3D). In the context of MIP, one usually utilizes NIP as the negative control. In solid-phase synthesis of MIP nanobodies, this approach is not feasible: synthesizing a particle on the surface requires a template; which, per definition, is absent in NIP. Therefore, one usually compares selectivities of two different MIP particles to assess selective binding. Figure 3D clearly demonstrates this: although NIP nanoparticles are more uniform in shape and morphology than the corresponding MIP, they are around 6–7 times larger than *S. epidermidis* cells. AFM images (Figure 4A,B) confirm the SEM data. As one can see, the MIP particles are uniform in diameter, which is around 107 nm. Overall, the particles are not only spherical, but also have a smooth outer surface [29,30]. 

The favorable size and positive zeta potential hence make protocol 3 the most useful for preparing high-affinity MIP nanoparticles despite the lowest overall yield.

### 3.2. Bacterial Cell Surface and Morphology Changes 

**Figure 5:**
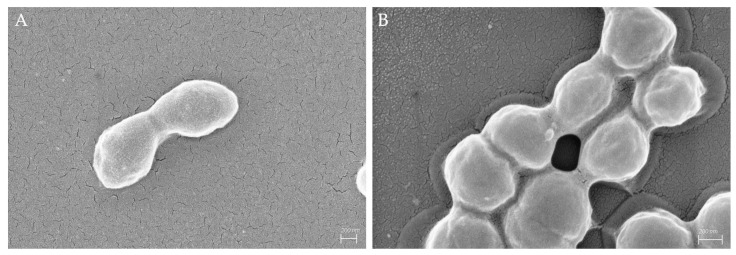
SEM images of native *S. epidermidis* (**A**); and *S. epidermidis* incubated with 50 mg/L. MIP nanoparticles overnight (**B**).

In the first step, it was necessary to characterize the surfaces of bacterial cells by SEM. Figure 5A reveals that native *S. epidermidis* cells are rather smooth. However, after incubating with MIP nanoparticles, the surface roughness of *S*. *epidermidis* seemingly does not change. One reason may be that it is not possible to extract height information from SEM images. (Figure 5B). Thus, SEM is not inherently suitable to assess structure heights on the bacteria: this requires AFM data.

### 3.3. AFM Characterization of Binding

**Figure 6:**
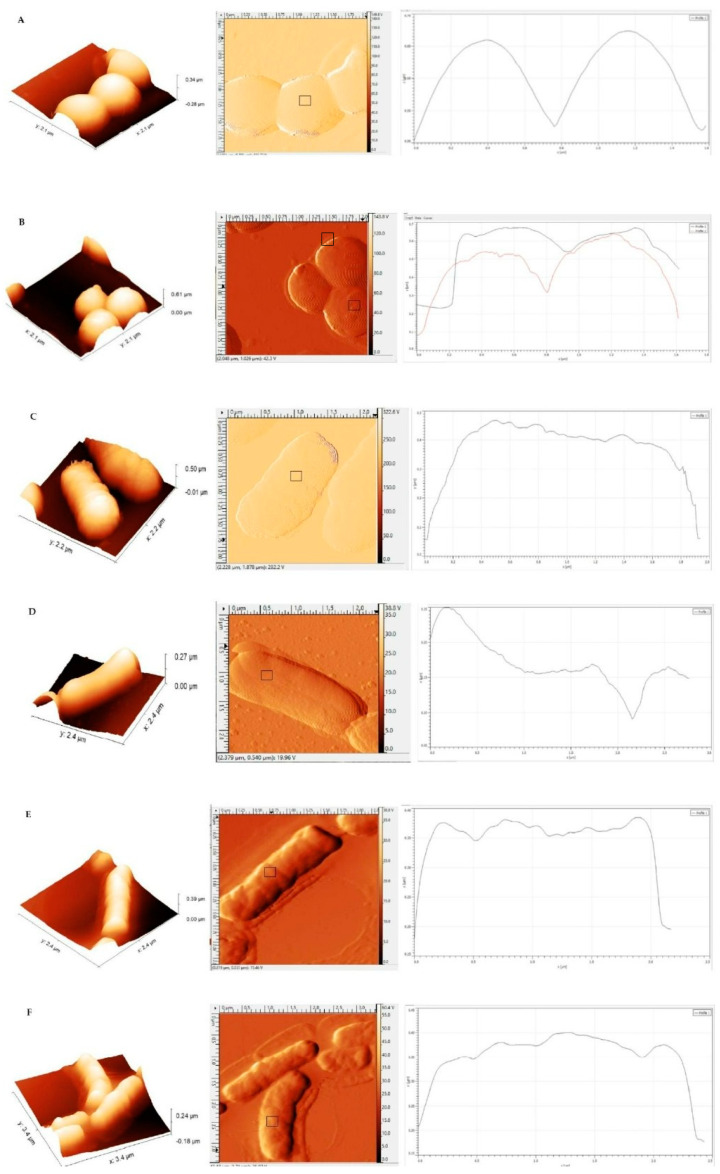
AFM 3 D image projections (left), AFM error images (middle) and AFM profile (right) *S. epidermidis* in the absence of the nanoparticles (**A**); *S. epidermidis* incubated overnight with 50 mg/L MIP nanoparticles (**B**); *E.coli* in the absence of the nanoparticles (**C**); *E.coli* incubated with 50 mg/L MIP nanoparticles (**D**); *B. subtilis* in the absence of the nanoparticles (**E**); *B. subtilis* incubated overnight with 50 mg/L MIP nanoparticles (**F**).

Figure 6 summarizes the AFM studies of binding between MIP nanoparticles and different bacteria species. For that purpose, it shows 3D images, error profiles, and a typical height image, respectively, for each experiment. This approach is in line with previous studies having demonstrated that changing surface roughness in AFM indicates binding between bacteria and nanoparticles [27,31,32]. Figure 6A shows that the surfaces of *S. epidermidis* are rather smooth. After incubating with MIP nanoparticles, the surface roughness increases, which becomes apparent especially in the height image (Figure 6B). One reason for such behavior may be the zeta potential of *S*. *epidermidis,* which is around −17 mV [33]. In contrast, the zeta potential of MIP nanoparticles is +8 mV as a consequence of including N-(3-aminopropyl) methacrylamide hydrochloride (APM) as a functional monomer. This increases affinity between the two species. It is known that this, among others, results in membrane depolarization and inhibition of bacterial growth [33]. However, for the other two species, i.e., *E. coli* (Figure 6C,D) and *B. subtilis,* the roughness does not change (Figure 6E,F).

This already strongly indicates that MIP nanoparticle ensembles synthesized on the surfaces of *B. subtilis* do bind to the surface of their respective template bacteria in a selective manner. However, it is of course necessary to assess this effect in a more quantitative manner. To achieve this, the best way is to calculate RMS roughness and Ra roughness of the cell surfaces of both control and treated bacteria, respectively, from the AFM images. Table 2 summarizes the corresponding data. 

**Figure 7:**
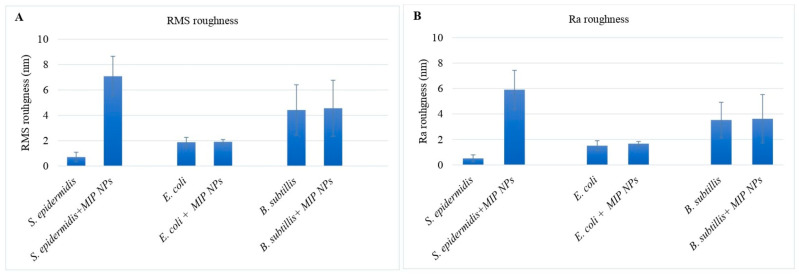
Surface roughness changes of *S. epidermidis, E. coli,* and *B. subtilis* after incubating with MIP nanoparticles. (**A**): RMS roughness; (**B**): Ra roughness.

The RMS average roughness of a surface is calculated from the roughness profile [34]. The surfaces of untreated *S. epidermidis*, *E. coli,* and *B. subtilis* cells, respectively, revealed values of 0.7 ± 0.3, 1.8 ± 0.3, and 4.4 ± 1.9 (Table 2), which is in good agreement with previous data [27,32]. Figure 7 shows the analysis of surface roughness of all selected bacteria, both in terms of RMS roughness (Figure 7A) and Ra roughness (Figure 7B). The results in Figure 7A clearly show that the surface roughness of *S. epidermidis* increases after exposing the bacteria to MIP nanoparticles. In contrast, *E. coli* and *B. subtilis* lead to different results: RMS roughness only slightly increased after incubating with MIP nanoparticles. The same can be said about Ra roughness, which describes the area between the roughness profile and its mean line, or the integral of the absolute value of the roughness profile height over the evaluation length [34]. The tendencies of Ra roughness and RMS roughness are consistent with each other.

Evidently, incubating *S. epidermidis* with MIP nanoparticles increases their surface roughness, whereas this effect is much weaker in the case of *E. coli* and *B. subtilis*. 

**Figure 8:**
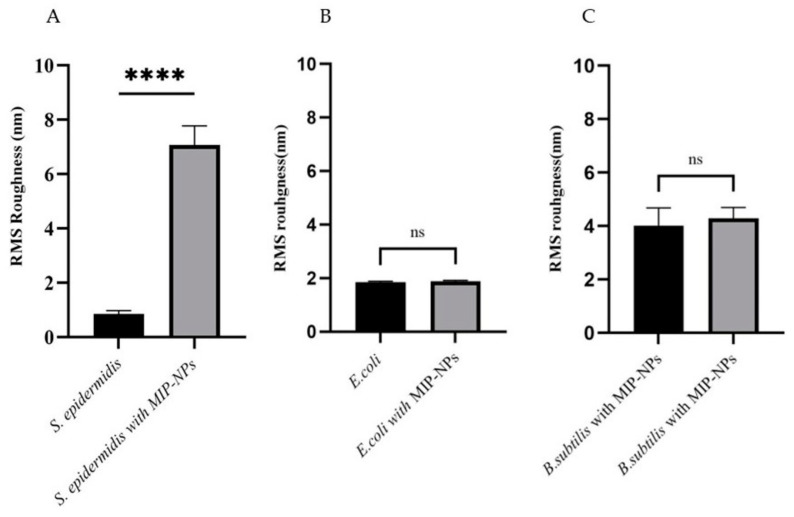
Analysis of the surface roughnesses (calculated based on the AFM images) of bacteria *S. epidermidis* (**A**)*, E. coli* (**B**)*,* and *B. subtilis* (**C**) in their native states (left-hand column) and incubated with 50 mg/L MIP nanoparticles, respectively, analyzed by unpaired t-test. The data represent mean ± standard deviation of roughness. **** *p* < 0.0001, ns = no-significant (*p* > 0.005).

However, it is necessary to establish by an unpaired t-test if these surface roughness changes are significant. The difference between *S. epidermidis* incubated with MIP nanoparticles and native, untreated cells is 6.224 ± 0.1398 nm (Figure 8A), which is significant. In contrast, *E. coli* and *B. subtilis* do not show significant changes (Figure 8B,C). 

### 3.4. Fluorescence Characterization of Binding

Though roughness analysis suggests selective binding between bacteria surfaces and particles, it is still somewhat generic: it relies on physical parameters of the respective surface, i.e., it does not contain explicit chemical information. To check those results in an independent manner, we carried out binding experiments between fluorescence-labelled bacteria and fluorescence-labelled MIP nanoparticles, respectively. 

**Figure 9:**
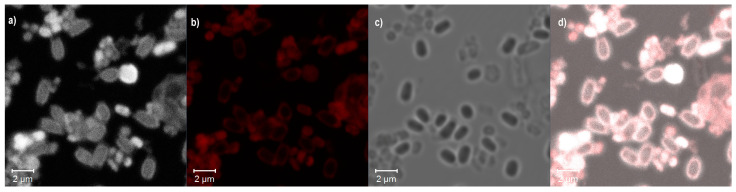
Fluorescence and light microscopy images of: (**a**) *S. epidermidis* cells on a surface; (**b**) MIP nanoparticles on the surface; (**c**) transmitted light image of cells plus nanoparticles; (**d**) overlay of transmitted light image and fluorescence data.

From the images in Figure 9, it becomes immediately clear that such binding indeed takes place: it shows different images of labelled *S. epidermidis* surfaces after incubating with MIP nanoparticles. Evidently, the areas overlap where both fluorescence labels—MemBrite and TRITC—emit (Figure 9a,b). They also clearly correlate with the presence of bacteria cells on the surface (Figure 9c). Figure 9d demonstrates this by combining all channels into one image. 

**Figure 10:**
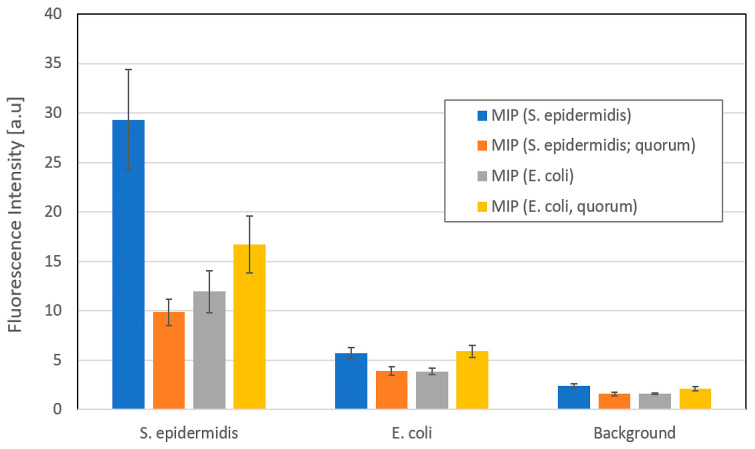
Fluorescence intensities obtained from exposing different bacteria strains to four different MIP. Intensities result from averaging the mean gray values of five areas of each combination of surface and MIP, respectively.

Figure 10 summarizes the data in a more quantitative manner, namely by assessing the fluorescence intensity in the gray channels of different spots. It also compares the MIP ensembles developed in this work with a material developed earlier by Piletsky et al. [26], who published some “snapshot imprinting” on cancer cell surfaces. Last, but not least, it compares particles synthesized on two different bacteria species, namely *S. epidermidis* and *E. coli*, respectively. Evidently, MIP NP ensembles synthesized on the surfaces of *S. epidermidis* preferably bind to that species with a wide margin: selectivity factors to *E. coli* are higher than five. This is the case for both types of NPs. However, the difference in data between the two different polymer types shows that it is imperative to optimize the monomer composition for each target analyte. The image is less clear for the MIP particles synthesized on the surfaces of *E. coli*. First, those polymers have, of course, not been optimized (the measurements are purely for reference reasons). Second, it is known that *S. epidermidis* strongly adheres to a wide variety of surfaces. Hence, one would also expect the opposite effect. Going into more detail in this regard, however, is beyond the scope of this paper. Finally, neither nanoparticle ensemble shows sizeable background signal, which denotes unspecific binding on the respective glass surface. Together, these data confirm that MIP nanoparticle ensembles are indeed a useful tool for targeting defined bacteria species, and thus allowing for identifying them in a rapid manner.

## 4. Conclusions

AFM and fluorescence results both indicate that the MIP nanoparticle ensembles selectively bind to the cell surfaces of *S. epidermidis.* The beauty of the approach for rapid bacteria identification lies in the fact that the exact binding sites on the respective cell surface may remain unknown: as long as the particle ensemble fits the chemical properties of the respective cell, it binds. In contrast to current standard methods for identifying bacteria, this is more straightforward because it requires neither amplification, such as in PCR, nor cell culturing on an agar plate. Of course, in the next step, it will be of interest to assess which types of molecules (saccharides, proteins, etc.) are responsible for such binding to better understand the intricacies of the binding behavior. 

## Figures and Tables

**Table 1:** Polymer compositions, zeta potentials, sizes, and high-affinity particle yields (with respect to overall amount of monomer) of MIP nanoparticles.

Protocol	NIPAM (mmol)	TBAM (mmol)	APM (mmol)	EBAM (mmol)	Zeta Pot mV	Size (nm)	Yield
1	0.66	-	-	0.06	−20.9	~200	~4.7%
2	0.6	0.06	-	0.06	−11.8	~50	~7.5%
3	0.54	0.06	0.06	0.06	+7.5	~70–110	~3.0%

**Table 2:** RMS roughness and Ra roughness of the AFM data in Figure 6.

Bacteria Cell	Native Bacteria		Bacteria Incubated with MIP Nanoparticles	
	RMS Roughness (nm)	Ra Roughness (nm)	RMS Roughness (nm)	Ra Roughness (nm)
*S. epidermidis*	0.7 ± 0.3	0.5 ± 0.2	7.0 ± 1.5	5.9 ± 1.5
*E. coli*	1. 8 ± 0.3	1.5 ± 0.3	1.9 ± 0.1	1.6 ± 0.1
*B. subtillis*	4.4 ± 1.9	3.5 ± 1.3	4.5 ± 2.2	3.6 ± 1.9

## Data Availability

Not applicable.

## References

[1] Cabrera J.N., Ruiz M.M., Fascio M., D’Accorso N., Mincheva R., Dubois P., Lizarraga L., Negri R.M. (2017). Increased Surface Roughness in Polydimethylsiloxane Films by Physical and Chemical Methods. Polymers.

[2] Fornazari F., Guimarães F., Teixeira C., Langoni H. (2012). Isolation of Staphylococcus epidermidis from inflamed upper respiratory tract of an orange-spined hairy dwarf porcupine (Sphiggurus villosus). J. Venom. Anim. Toxins Incl. Trop. Dis..

[3] Huang T.Y., Jiang Y.E., Scott D.A. (2022). Culturable bacteria in the entire acne lesion and short-chain fatty acid metabolites of Cutibacterium acnes and Staphylococcus epidermidis isolates. Biochem. Biophys. Res. Commun..

[4] Fernández-Rodríguez D., Colín-Castro C.A., Hernández-Durán M., López-Jácome L.E., Franco-Cendejas R. (2021). Staphylococcus epidermidis small colony variants, clinically significant quiescent threats for patients with prosthetic joint infection. Microbes Infect..

[5] Yogo A., Yamamoto S., Sumiyoshi S., Iwamoto N., Aoki K., Motobayashi H., Tochitani K., Shimizu T., Murashima T., Nishikawa N. (2022). Two cases of pyelonephritis with bacteremia by Staphylococcus epidermidis in male patients with nephrolithiasis: Case reports and a literature review. J. Infect. Chemother..

[6] Rupp M.E., Fey P.D. (2010). Staphylococcus epidermidis and Other Coagulase-Negative Staphylococci.

[7] Pagano L., Gkartziou F., Aiello S., Simonis B., Ceccacci F., Sennato S., Ciogli A., Mourtas S., Spiliopoulou I., Antimisiaris S.G. (2022). Resveratrol loaded in cationic glucosylated liposomes to treat Staphylococcus epidermidis infections. Chem. Phys. Lipids.

[8] Jukes L., Mikhail J., Bome-Mannathoko N., Hadfield S.J., Harris L., El-Bouri K., Davies A., Mack D. (2010). Rapid differentiation of Staphylococcus aureus, Staphylococcus epidermidis and other coagulase-negative staphylococci and meticillin susceptibility testing directly from growth-positive blood cultures by multiplex real-time PCR. J. Med Microbiol..

[9] Malik S., Peng H., Barton M. (2005). Antibiotic resistance in staphylococci associated with cats and dogs. J. Appl. Microbiol..

[10] A Hossain M., Bilkis L., Musa A.K., Mahamud C., Bari M.S., Haque N., Muhammad N., Parvin U.S., Islam M.T., I Khan S. (2009). Antibiotic susceptibility pattern of Staphylococcus epidermidis. Mymensingh Med. J..

[11] Shin J.H., Ranken R., Sefers S.E., Lovari R., Quinn C.D., Meng S., Carolan H.E., Toleno D., Li H., Lee J.N. (2013). Detection, Identification, and Distribution of Fungi in Bronchoalveolar Lavage Specimens by Use of Multilocus PCR Coupled with Electrospray Ionization/Mass Spectrometry. J. Clin. Microbiol..

[12] Albert M., Friedrich J., Adhikari N., Day A., Verdant C., Heyland D.K. (2008). Utility of Gram stain in the clinical management of suspected ventilator-associated pneumonia: Secondary analysis of a multicenter randomized trial. J. Crit. Care.

[13] Ruzicka F., Hola V., Votava M., Tejkalová R., Horvát R., Heroldová M., Woznicová V. (2004). Biofilm detection and the clinical significance ofStaphylococcus epidermidis isolates. Folia Microbiol..

[14] Pereira E.M., Schuenck R.P., Malvar K.L., Iorio N.L., Matos P.D., Olendzki A.N., Oelemann W.M., dos Santos K.R. (2010). Staphylococcus aureus, Staphylococcus epidermidis and Staphylococcus haemolyticus: Methicillin-resistant isolates are detected directly in blood cultures by multiplex PCR. Microbiol. Res..

[15] Hoshino Y., Koide H., Urakami T., Kanazawa H., Kodama T., Oku N., Shea K.J. (2010). Recognition, Neutralization, and Clearance of Target Peptides in the Bloodstream of Living Mice by Molecularly Imprinted Polymer Nanoparticles: A Plastic Antibody. J. Am. Chem. Soc..

[16] Suedee R., Seechamnanturakit V., Canyuk B., Ovatlarnporn C., Martin G.P. (2006). Temperature sensitive dopamine-imprinted (N,N-methylene-bis-acrylamide cross-linked) polymer and its potential application to the selective extraction of adrenergic drugs from urine. J. Chromatogr. A.

[17] Bräuer B., Thier F., Bittermann M., Baurecht D., Lieberzeit P.A. (2021). Raman Studies on Surface-Imprinted Polymers to Distinguish the Polymer Surface, Imprints, and Different Bacteria. ACS Appl. Bio Mater..

[18] Sharif H.E., Dennison S.R., Tully M., Crossley S., Mwangi W., Bailey D., Graham S., Reddy S.J. (2022). Evaluation of electropolymerized molecularly imprinted polymers (E-MIPs) on disposable electrodes for detection of SARS-CoV-2 in saliva. Anal. Chim. Acta.

[19] Idil N., Mattiasson B. (2017). Imprinting of Microorganisms for Biosensor Applications. Sensors.

[20] Liu F., Liu X., Ng S.-C., Chan H.S.-O. (2006). Enantioselective molecular imprinting polymer coated QCM for the recognition of l-tryptophan. Sensors Actuators B: Chem..

[21] Gao F.-X., Ma X.-T., He X.-W., Li W.-Y., Zhang Y.-K. (2013). Smart surface imprinting polymer nanospheres for selective recognition and separation of glycoprotein. Colloids Surf. A: Physicochem. Eng. Asp..

[22] Pan J., Xue X., Wang J., Xie H., Wu Z. (2009). Recognition property and preparation of Staphylococcus aureus protein A-imprinted polyacrylamide polymers by inverse-phase suspension and bulk polymerization. Polymer.

[23] Canfarotta F., Poma A., Guerreiro A., Piletsky S. (2016). Solid-phase synthesis of molecularly imprinted nanoparticles. Nat. Protoc..

[24] Rangel P.X.M., Laclef S., Xu J., Panagiotopoulou M., Kovensky J., Bui B.T.S., Haupt K. (2019). Solid-phase synthesis of molecularly imprinted polymer nanolabels: Affinity tools for cellular bioimaging of glycans. Sci. Rep..

[25] Dar K.K., Shao S., Tan T., Lv Y. (2020). Molecularly imprinted polymers for the selective recognition of microorganisms. Biotechnol. Adv..

[26] Piletsky S.S., Piletska E., Poblocka M., Macip S., Jones D.J., Braga M., Cao T.H., Singh R., Spivey A.C., Aboagye E.O. (2021). Snapshot imprinting: Rapid identification of cancer cell surface proteins and epitopes using molecularly imprinted polymers. Nano Today.

[27] Maillard A.P.F., Gonçalves S., Santos N.C., de Mishima B.A.L., Dalmasso P.R., Hollmann A. (2019). Studies on interaction of green silver nanoparticles with whole bacteria by surface characterization techniques. Biochim. Biophys. Acta (BBA) Biomembr..

[28] Zhang Y., Fu T., Cui K., Shen F., Wang J., Yu L., Mao H. (2021). Evolution of surface morphology, roughness and texture of tungsten disilicide coatings on tungsten substrate. Vacuum.

[29] Choi S., Jung G.B., Kim K.S., Lee G.-J., Park H.-K. (2014). Medical applications of atomic force microscopy and Raman spectroscopy. J. Nanosci. Nanotechnol..

[30] Sajini T., Mathew B. (2021). A brief overview of molecularly imprinted polymers: Highlighting computational design, nano and photo-responsive imprinting. Talanta Open.

[31] Kumar B.R., Rao T.S. (2012). AFM studies on surface morphology, topography and texture of nanostructured zinc aluminum oxide thin films. Dig. J. Nanomater. Biostructures.

[32] Liu S., Ng A.K., Xu R., Wei J., Tan C.M., Yang Y., Chen Y. (2010). Antibacterial action of dispersed single-walled carbon nanotubes on Escherichia coli and Bacillus subtilis investigated by atomic force microscopy. Nanoscale.

[33] Ong T.H., Chitra E., Ramamurthy S., Ling C.C.S., Ambu S.P., Davamani F. (2019). Cationic chitosan-propolis nanoparticles alter the zeta potential of S. epidermidis, inhibit biofilm formation by modulating gene expression and exhibit synergism with antibiotics. PLoS ONE.

[34] Krishnan B.R. (2020). Review of surface roughness prediction in machining process by using various parameters. Int. J. Recent Trends Eng. Res..

